# Nutrition behaviour change communication causes sustained effects on IYCN knowledge in two cluster‐randomised trials in Bangladesh

**DOI:** 10.1111/mcn.12498

**Published:** 2017-08-07

**Authors:** John Hoddinott, Akhter Ahmed, Naureen I. Karachiwalla, Shalini Roy

**Affiliations:** ^1^ Division of Nutrition Sciences Cornell University Ithaca New York USA; ^2^ Poverty, Health, and Nutrition Division International Food Policy Research Institute (IFPRI) Dhaka Bangladesh; ^3^ Poverty, Health, and Nutrition Division International Food Policy Research Institute (IFPRI) Washington District of Columbia USA

**Keywords:** Bangladesh, behaviour change communication, infant and child nutrition, IYCN knowledge, low‐income countries, persistence

## Abstract

Behaviour change communication (BCC) can improve infant and young child nutrition (IYCN) knowledge, practices, and health outcomes. However, few studies have examined whether the improved knowledge persists after BCC activities end. This paper assesses the effect of nutrition sensitive social protection interventions on IYCN knowledge in rural Bangladesh, both during and after intervention activities. We use data from two, 2‐year, cluster randomised control trials that included nutrition BCC in some treatment arms. These data were collected at intervention baseline, midline, and endline, and 6–10 months after the intervention ended. We analyse data on IYCN knowledge from the same 2,341 women over these 4 survey rounds. We construct a number correct score on 18 IYCN knowledge questions and assess whether the impact of the BCC changes over time for the different treatment groups. Effects are estimated using ordinary least squares accounting for the clustered design of the study. There are 3 main findings: First, the BCC improves IYCN knowledge substantially in the 1st year of the intervention; participants correctly answer 3.0–3.2 more questions (36% more) compared to the non‐BCC groups. Second, the increase in knowledge between the 1st and 2nd year was smaller, an additional 0.7–0.9 correct answers. Third, knowledge persists; there are no significant decreases in IYCN knowledge 6–10 months after nutrition BCC activities ended.

**Abbreviations**
BCC: behaviour change communication
CNW: community nutrition worker
ESDO: Eco‐Social Development Organization
FFO: field facilitating officer
IYCN: infant and young child nutrition
IFPRI: International Food Policy Research Institute
RCT: randomised control trial
TMRI: Transfer Modality Research Initiative
WFP: World Food Programme

## 1 INTRODUCTION

Chronic undernutrition is widespread in many low‐income countries (Black et al., 2013), including in Bangladesh, where 36% of children under 5 years old are stunted (NIPORT, 2015). Inadequate infant and young child nutrition (IYCN) knowledge and practices lead to poorer preschool nutrition outcomes, and subsequently, to poorer health, education, and labour outcomes in adulthood (Ahmed et al., 2012; Avula et al., 2013; Black et al., 2013; Hoddinott et al., 2013; World Health Organization, 2008). Correct IYCN knowledge strongly predicts appropriate IYCN practices (Balogun, Dagvadorj, Anigo, Ota, & Sasaki, 2015; Stewart, Iannotti, Dewey, Michaelsen, & Onyango, 2013; Tuan, Nguyen, Hajeebhoy, & Frongillo, 2014; Yanikkerem, Tuncer, Yilmaz, Aslan, & Karadeniz, 2009). As such, much research and policy has been dedicated to assessing methods by which to improve IYCN knowledge and practices. Behaviour change communication (BCC) has been shown to improve IYCN knowledge, with improvements also seen in IYCN practices, and to some extent, health outcomes (Bhutta et al., 2008; Caulfield, Huffman, & Piwoz, 1999; Dewey & Adu‐Afarwuah, 2008; Imdad, Yakoob, & Bhutta, 2011; Shi & Zhang, 2011).

An under‐researched question, particularly for intervention design, is the appropriate duration of BCC and whether knowledge gained from BCC persists. This paper addresses these questions, drawing on data from two cluster randomised control trials (RCTs) fielded in Bangladesh that included BCC as part of some, but not all, treatment arms. IYCN knowledge was measured at baseline, after 1 year and after 2 years of intensive BCC, and 6–10 months after the BCC ended. These data, along with the randomised design, allow us to estimate the impact of the BCC intervention on the treatment group after 1 and 2 years, as well as whether these changes in knowledge are sustained after the intervention ends.

**Key messages**

- This study documents the effect over time of a behaviour change communication (BCC) intervention on infant and young child nutrition knowledge among mothers in Bangladesh.
- BCC results in improved knowledge of infant and young child nutrition, and this gain in knowledge persists 6–10 months after BCC activities end.
- Much of the gain in knowledge is achieved in the first 12 months of training.
- Impacts of BCC programmes may be underestimated if persistence of effects is unaccounted for.

## 2 METHODS

### 2.1 Programme description

The Transfer Modality Research Initiative (TMRI) was conducted between March 2012 and May 2014. Two RCTs were conducted in rural areas: one in the northwest region of Bangladesh (the “north”), where poverty and insecurity rates are high but food markets function well, and one in the coastal southern region of Bangladesh (the “south”), where poverty is slightly lower than in the north, climate shocks are more frequent, and although food availability is similar to the north, food markets are less accessible at certain times of the year. In the north, study villages were randomly assigned to a control group or one of four treatment arms in which beneficiaries received a cash transfer (“Cash”), a food ration (“Food”), a half cash transfer and half food ration (“Cash&Food”), or a cash transfer along with nutrition BCC (“Cash + BCC”). In the south, study villages were also randomly assigned to a control group or one of four treatment arms; the first three treatment groups were the same as in the north. The final treatment group in the south was different: Instead of a cash transfer along with nutrition BCC, beneficiaries received a food ration along with nutrition BCC (“Food + BCC”). All beneficiaries were poor households with a child aged 0–24 months in March 2012. Transfer payments were made for 24 months.

The programme was designed and evaluated by the International Food Policy Research Institute and implemented by the United Nations' World Food Programme (WFP). WFP managed the procurement and delivery of transfers, as well as the nutrition BCC training, and routinely monitored the programme. A nongovernmental organization contracted by WFP, the Eco‐Social Development Organization (ESDO), was responsible for the field implementation of project activities, including distributing the monthly food and cash transfers, delivering the nutrition BCC, and performing reporting activities.

Beneficiaries in the “Cash” arms received a monthly payment of 1,500 Taka (approximately 19 USD) per household. Beneficiaries in the “Food” arms received a monthly food ration of 30 kg of rice, 2 kg of *mosoor* pulse (a lentil), and 2 L of micronutrient‐fortified cooking oil. These quantities were chosen so that the initial value of the food ration was equal to the value of the cash transfer of the beneficiaries in the “Cash” treatment arms. Beneficiaries in the “Cash&Food” treatment arms received 750 Taka, 15 kg of rice, 1 kg of *mosoor* pulse, and 1 L of micronutrient‐fortified cooking oil; half of each of the two types of transfers. All transfers were made to mothers.

The beneficiaries of the “Cash + BCC” arm in the north and of the “Food + BCC” arm in the south received the same transfer as in the “Cash” only and “Food” only treatment groups, as well as a suite of intensive nutrition BCC interventions. The main intervention was a 1‐hr group session held weekly in the village in which participants resided, led by a trained community nutrition worker (CNW). The group sessions covered the following topics: nutrition, diet diversity, and health; handwashing, hygiene, and health; diet diversity and micronutrients; breastfeeding; complementary foods for children 6–24 months; feeding and treatment of children with diarrhoea; maternal nutrition; encouraging homestead food production; and women's status and relationships with influential family members such as husbands and mothers‐in‐law (e.g., negotiating intrahousehold relationships related to feeding preschool children). The sessions used a variety of methods including question and answer, presentations, practical demonstrations, role playing, and interactive call and answer. Some sessions were held exclusively for beneficiaries; in other sessions, husbands, mothers‐in‐law, and other influential individuals from beneficiaries' homes were also encouraged to attend. The husband, being the child's father and also responsible for both food purchases and other decision‐making, was considered an important figure for supporting the uptake of the IYCF lessons. Similarly, mothers‐in‐law play an important role in child‐rearing in Bangladesh and would also influence the uptake of lessons learned.

The BCC session materials were derived from material developed for Alive & Thrive (A&T) in Bangladesh, a comprehensive programme aimed at improving breastfeeding and complementary feeding practices and ultimately reducing stunting and anaemia among young children. This curriculum has been used widely throughout Bangladesh (Hoddinott, Karachiwalla, Ledlie, & Roy, 2016; Nguyen et al., 2014). A&T followed World Health Organization and United Nations Children's Fund guidelines for IYCN. A&T drew on a variety of methods to design the programme, including several behaviour change theories (these theories—the theory of “reasoned action”; models focused on interpersonal interactions, self‐efficacy, and learning from role models; and community models emphasising the diffusion of information through social networks—were combined to produce the socioecological model that guided their programme design), quantitative and qualitative formative research, trials of improved practice, previous studies in other countries, assessments of media habits, and stakeholder consultations (Baker, Sanghvi, Hajeebhoy, Martin, & Lapping, 2013). As in A&T, the BCC in TMRI focused on the first 2 years of life. TMRI also followed A&T in terms of the content of the BCC sessions and an approach that included community engagement, group BCC sessions, and home visits (Baker et al., 2013; Haider et al., 2010). Because many factors underlying the IYCN recommendations—including local food availability and seasonality—are comparable in the north and south of Bangladesh, TMRI used the same approach to the BCC across the two regions.

CNWs also conducted home visits to beneficiaries to follow up on topics that were discussed in the group sessions, as well as to answer any questions or concerns that beneficiaries may have had. Attendance at the nutrition BCC sessions was a soft condition of receipt of the transfers. When a mother missed a session, a CNW would follow up with a home visit to uncover what the reason was for missing the session, and no beneficiaries were dropped from the study for failing to attend sessions. The nutrition BCC group sessions were carried out for 2 years, from May 2012 to April 2014.

The ESDO, the WFP, and the study team conducted home visits and interviews on the nutrition BCC to identify any problems with delivery and to implement any solutions needed throughout the intervention period. Further, CNWs and ESDO staff held community meetings and met with influential members of study villages to explain the purpose of the training and to provide them with the information being conveyed to study participants. CNWs received training before the intervention began, and refresher training was also provided after both three and 12 months after the start of the intervention.

Qualitative and quantitative monitoring data were collected throughout the intervention period, and results indicated that the TMRI was implemented as designed. Of households receiving the nutrition BCC intervention, households in the north attended approximately 48 of the scheduled 52 sessions per year on average, and households in the south attended approximately 49 of the scheduled 52 sessions per year on average. Each session lasted approximately 1 hr, on average. Eighty‐three percent (556/670) of respondents reported that if a session was missed, the CNW followed up with an in‐home visit. Further, CNWs did acquire the knowledge on which they were trained: A CNW survey that took place in April 2014 conducted a quiz on the key nutrition messages, which CNWs were to convey to beneficiaries as part of the nutrition BCC. Of the 14 questions asked, the mean score was 13.2 (*SD* 0.80) in the north and was 13.5 (*SD* 1.00) in the south.

### 2.2 Study design

The survey instrument was piloted among 120 households in 20 villages that were not part of the study before the start of the survey in February 2012. Questions were written in Bangla and read aloud. Questions were originally drafted in English, translated to Bangla, and then back‐translated into English to ensure validity. The survey was administered by DATA, a survey company with 23 years of experience in carrying out large‐scale household surveys across Bangladesh. The study included four rounds of quantitative data collection: a baseline survey carried out from March to April 2012, before the interventions began in May 2012, a midline survey carried out in June 2013, an endline survey in April 2014, just before the interventions ended that month, and a post‐endline survey from October 2014 to February 2015, 6 to 10 months after the intervention ended. Data used in this study were collected from the same respondents over time, forming a panel survey. Although the baseline, midline, and endline surveys included respondents from all eight treatment groups (four in the north and four in the south) and the two control arms, the post‐endline survey only included respondents from the “Cash,” “Cash + BCC,” and control arms in the north and from the “Food,” “Food + BCC,” and control arms in the south. Budget constraints precluded collecting post‐endline data from other treatment arms.

In the baseline, midline, and endline rounds, questionnaires were administered to both the head of the household and to the primary female caregiver (usually the mother) of the “index” child, defined to be a child in the household aged 0–24 months in March 2012. If there was more than one child in the household aged 0–24 months, the youngest child was selected as the index child. Male enumerators conducted surveys with male respondents, and female enumerators conducted surveys with female respondents. Questions were asked on topics regarding household demographics and housing quality, food consumption and dietary diversity, and knowledge of IYCN practices. The same IYCN knowledge questions were asked in each round, and attempts were made to have the same respondent answer the IYCN knowledge questions each time. The questions were asked in the same order to all respondents in all rounds. Respondents were not provided with feedback as to the correct answers.

The post‐endline round was designed for a component of the study assessing early childhood development of the “index” child. It was conducted in a village centre (such as a community club or a school) where caregivers brought their children for early childhood development testing. A short instrument including the same IYCF knowledge questions was administered to caregivers.

The study is registered with http://ClinicalTrials.gov (ID: NCT02237144). The study was conducted according to the guidelines of the Declaration of Helsinki, and the study received ethical approval from the Institutional Review Board of the International Food Policy Research Institute, Washington, DC. The study was also reviewed by the Ministry of Food and Disaster Management in Bangladesh, who issued authorisation letters to conduct the surveys. Consent to participate in the study was received verbally from respondents, and this verbal consent was witnessed and formally recorded.

### 2.3 Sample design

The TMRI study used a cluster RCT design. Separate sampling processes were followed in the north and in the south. In each region, five sub‐districts (*upazilas*) were selected from a list of *upazilas* where, in 2010, the proportion of households living below the lower poverty line in Bangladesh was 25% or more (World Bank, World Food Programme; Bangladesh Bureau of Statistics, 2010). All villages within these five *upazilas* were listed. Both villages with fewer than 125 households and villages that were considered peri‐urban were dropped. Simple random sampling was used to select villages from this list. Using a random number generator, each village was assigned a random number. Then, villages were sorted in ascending numerical order, and the first 275 villages were retained. The first 50 villages in this sorted list were assigned to the “Cash” group, the next 50 villages were assigned to the “Food” group, the next 50 villages were assigned to the “Cash&Food” group, the next 50 villages were assigned to the “Cash + BCC” group in the north and to the “Food + BCC” group in the south, and the next 50 villages were assigned to the “Control” group. The remaining 25 villages were held as a reserve. In the 250 selected villages, a village census was carried out, which collected information on household demographics, poverty indicators, and whether households were participating in social safety net and other targeted interventions. From these data, a list of households was constructed that were considered poor (poverty status was based on a score calculated using information on the age and education of the household head, housing characteristics, ownership of consumer durables, land ownership, and household livelihoods), would have a child aged 0–24 months by the time the intervention began, and were not receiving benefits from any other social safety net interventions. These were the eligible households for participation in the study. From each village, 10 households meeting these three conditions were randomly selected using simple random sampling and giving a total sample size of 5,000 targeted households.

Because in this analysis we focus on persistence of knowledge over the four survey rounds, we restrict the sample to the treatment arms that were covered by the post‐endline survey: namely, the control groups in both the north and the south, the “Cash” and “Cash + BCC” treatment arms in the north, and the “Food” and “Food + BCC” arms in the south. Among this sample of 3,000 households at baseline, 2,749 households were successfully re‐interviewed at post‐endline. Attrition between baseline and post‐endline was not systematically correlated with intervention arm or baseline characteristics. In both the north and the south, neither participation in any of the treatment groups nor the control groups affects attrition. In the north, only one variable (household assets) is statistically significantly correlated with attrition; however, the coefficient is extremely small (less than one thousandth of a point). In the south as well, there is only one statistically significant correlate of attrition: the age of the caregiver of the index child. This coefficient is also extremely small (a difference of 0.002 years). Because these are extremely small differences, we do not believe that attrition would bias our results. Within the post‐endline sample, 2,341 households had the same respondent for the IYCN knowledge questions in all four rounds of the survey. This includes 1,213 households in the north and 1,128 households in the south.

We calculated the ex‐post statistical power required for the outcomes specific to this paper. Using a significance level of 0.05, setting statistical power at 0.80, and using outcome‐specific means, standard deviations, and intra‐cluster correlations, a sample that includes 50 clusters per treatment arm and 10 households per cluster provides sufficient statistical power to detect an increase of 0.455 increase in the number of IYCN questions answered correctly.

### 2.4 Measures

In each survey round, there are 18 questions relating to IYCN knowledge that are asked of the caregiver of the index child. The questions are based on the material that was taught to participants in the BCC sessions and are listed in Table S1. Each question had multiple choice responses that were read to respondents. More than one response could be considered correct, and this was reflected in the scoring. We construct a “total knowledge score” comprising the number of questions answered correctly, which ranges from 0 to 18. We also construct three additional measures: one comprising the number of correct answers to the three breastfeeding questions (the “breastfeeding knowledge score”); one comprising the number of correct answers to the 10 micronutrients questions (the “micronutrients knowledge score”); and one comprising the number of correct answers to five questions on water, sanitation, and hygiene (the “WASH score”).

### 2.5 Statistical analysis

All statistical analyses were conducted using STATA 14 (StatCorp LP). We conduct the analysis separately for the north and for the south.

We first present descriptive statistics by region on the knowledge scores and other key household and respondent indicators at baseline, comparing the different treatment arms to assess baseline balance. We present means for each treatment group, as well as *p* values for *t* tests of the differences between treatment groups. Variables are considered balanced if *p* > .05. To show patterns over time, we then present figures showing the average total knowledge score in each of the four survey rounds for each of the three treatment groups, separately for the north and for the south.

We use analysis of covariance to measure the effect of treatment on knowledge across rounds (McKenzie, 2012). By conditioning on the respondent's baseline total knowledge score, a variable correlated with our outcome of interest, as McKenzie (2012) notes, we reduce the variance of the treatment estimator. Separately for the north and for the south, we run three ordinary least squares regressions: one with the midline total knowledge score as the outcome, one with the endline total knowledge score as the outcome, and one with the post‐endline total knowledge score as the outcome. All regressions are restricted to the sample for which the same respondent answered the IYCN knowledge questions in all four rounds. In addition to the baseline total knowledge score, we include dummy variables for the “Cash” and “Cash + BCC” treatment groups in the north and for the “Food” and “Food + BCC” treatment groups in the south. In both the north and the south, the “Control” group is the baseline category. Standard errors are clustered at the village level to account for the cluster randomised design of the study. Impacts are considered statistically significant if *p* ≤ .05.

We use chi‐squared tests to test whether the coefficients on the treatment arm dummy variables are statistically different from one another across regressions (comparing midline to endline and comparing endline to post‐endline). We use seemingly unrelated estimation, whereby parameter estimates and covariance matrices are stored into one parameter vector and simultaneous covariance matrix (Clogg, Petkova, & Haritou, 1995), and report the *p* values for tests that a coefficient in one regression is statistically equivalent to the same coefficient in a different regression. For example, we test whether the coefficient for the impact on knowledge at midline for the “Cash” group is the same as the coefficient for the impact on knowledge at endline for the “Cash” group. We do the same for the “Cash + BCC” in the north and for the “Food” and “Food + BCC” groups in the south. Coefficients are considered statistically different if *p* < .05.

We conduct the following robustness checks. We re‐estimate the impacts using the “breastfeeding knowledge” score, the “micronutrients knowledge” score, and the “WASH knowledge” score as outcomes instead, in order to assess whether persistence differs for these different types of questions. We re‐estimate the regressions for the total knowledge score outcomes at midline, endline, and post‐endline including baseline demographic variables as control variables to account for any imbalance at baseline. We conduct heterogeneity analysis to ascertain whether persistence between endline and post‐endline differs by various household characteristics. We look at differences in the age of the respondent for the IYCN knowledge questions, whether the respondent has had no formal education, the age of the youngest child in the household, and the number of days between the approximate end of the BCC (May 30, 2014) and the date of administration of the post‐endline survey. We conduct this heterogeneity analysis by including a control variable for the aspect of heterogeneity in which we are interested in the regression and interacting the control variable with the two treatment group dummy variables.

## 3 RESULTS

### 3.1 Characteristics of the study sample

Table 1 describes the baseline characteristics of the households and of the respondents answering the IYCN knowledge questions in the north (top panel) and in the south (bottom panel). Average household size is 4.98 (*SD* 1.39). The value of total household assets (including both consumer durables and productive assets) is approximately 16,320 (*SD* 22,670) Taka (about 200 USD in April 2012), with households in the south having slightly higher values of assets than households in the north. The average age of the head of the household is 35 (*SD* 9.84) years old. Few heads of household are female, only 9% (*n* = 220). However, there are more female‐headed households in the south than in the north. Sixty‐one percent (*n* = 1,440) of household heads have no schooling, and household heads in the north are less likely to have had schooling. The average age at baseline of the study's index child is 8 (*SD* 12.99) months. The average age at baseline of the youngest child in the household is 7.3 (*SD* 7.12) months. Forty‐three percent (*n* = 1,007) of the caregivers of the index children have no schooling, and the caregivers of index children in the north are less likely to have had any schooling. The average age of the caregiver of the index child is 27 (*SD* 6.49) years old. On average, the respondent for the IYCN knowledge questions got 8.59 (*SD* 2.30) out of all 18 questions correct, 1.53 (*SD* 0.67) out of 3 breastfeeding questions correct, 5.52 (*SD* 1.76) out of 10 micronutrient questions correct, and 1.53 (*SD* 0.67) out of 5 WASH questions correct at baseline. The statistics for the knowledge questions are very similar between the north and the south.

**Table 1:** Baseline means and balance of sample variables by intervention arm

North (*n* = 1,213)	*M*s			*p* values of differences		
	Cash	Cash + BCC	Control	Cash − Cash + BCC	Cash − Control	Cash + BCC − Control
Household characteristics						
Number of household members	4.78	4.84	4.78	.53	.99	.53
Value of total household assets	15,811.05	15,203.62	14,087.61	.71	.23	.42
Age of the head of household	35.32	35.42	35.59	.89	.71	.81
Head of household is female	0.07	0.07	0.06	.67	.89	.58
Head of the household has no schooling	0.69	0.69	0.70	.96	.64	.68
Age of the sample index child	8.43	8.20	8.14	.82	.76	.95
Age of the youngest child in the household	7.40	7.66	7.46	.62	.91	.69
Characteristics of the caregiver of the index child						
Caregiver of the index child has no schooling	0.50	0.52	0.49	.65	.83	.51
Age of the caregiver of the index child	27.30	28.03	27.70	.11	.40	.47
Number correct on all IYCN knowledge questions	8.79	8.77	8.62	.87	.27	.33
Number correct on breastfeeding questions	1.56	1.51	1.51	.35	.30	.94
Number correct on micronutrient questions	5.65	5.56	5.41	.43	.05	.21
Number correct on WASH questions	1.59	1.70	1.70	.24	.22	.99

South (*n* = 1,128)	*M*s			*p* values of differences		
	Food	Food + BCC	Control	Food − Food + BCC	Food − Control	Food + BCC − Control
Household characteristics						
Number of household members	5.24	4.97	5.27	.01	.76	.00
Value of total household assets	16,276.71	18,260.58	18,576.42	.26	.17	.86
Age of the head of household	35.71	34.09	35.13	.02	.41	.12
Head of household is female	0.14	0.12	0.10	.59	.15	.38
Head of the household has no schooling	0.56	0.50	0.53	.09	.34	.47
Age of the sample index child	7.63	8.10	7.57	.64	.95	.52
Age of the youngest child in the household	6.66	7.38	7.20	.17	.28	.73
Characteristics of the caregiver of the index child						
Caregiver of the index child has no schooling	0.40	0.33	0.33	.05	.06	.94
Age of the caregiver of the index child	28.04	27.42	27.93	.17	.82	.26
Number correct on all IYCN knowledge questions	8.45	8.45	8.43	.99	.90	.90
Number correct on breastfeeding questions	1.58	1.47	1.58	.03	.95	.03
Number correct on micronutrient questions	5.44	5.63	5.45	.18	.94	.21
Number correct on WASH questions	1.43	1.36	1.40	.39	.69	.64

*Note*. “Cash” (“Food”) refers to treatment arm in which a cash (food) transfer was provided to the participant household. “Cash + BCC” (“Food + BCC”) refers to treatment arm in which a cash (food) transfer, along with BCC training was provided to the participant household. “Control” refers to a treatment arm in which no benefits were provided to the participant household. Restricted to sample with same respondent over all four rounds of data collection. BCC = behaviour change communication; IYCN = infant and young child nutrition.

We also assessed whether the sample was balanced across these baseline characteristics. There are no significant differences in household or IYCN knowledge respondent characteristics between the three groups in the north. In the south, there are six significant differences: The number of household members in the “Food + BCC” group is slightly higher than in the other two groups, the proportion of caregivers with no schooling is slightly higher in the “Food” group, the number of breastfeeding questions answered correctly is slightly lower in the “Food + BCC” group compared to the other two groups, and the age of the household head is slightly lower in the “Food + BCC” group compared to the “Food” group.

### 3.2 Persistence of IYCN knowledge caused by the programme

**Figure 1:**
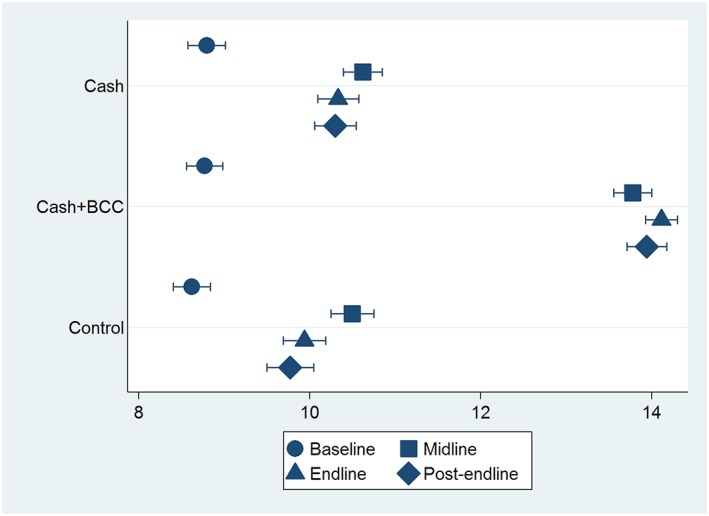
Infant and young child nutrition knowledge scores over survey rounds and by treatment groups—north. Knowledge scores include 18 questions on breastfeeding, sanitation, and other health and nutrition topics. Bars represent 95% confidence intervals

**Figure 2:**
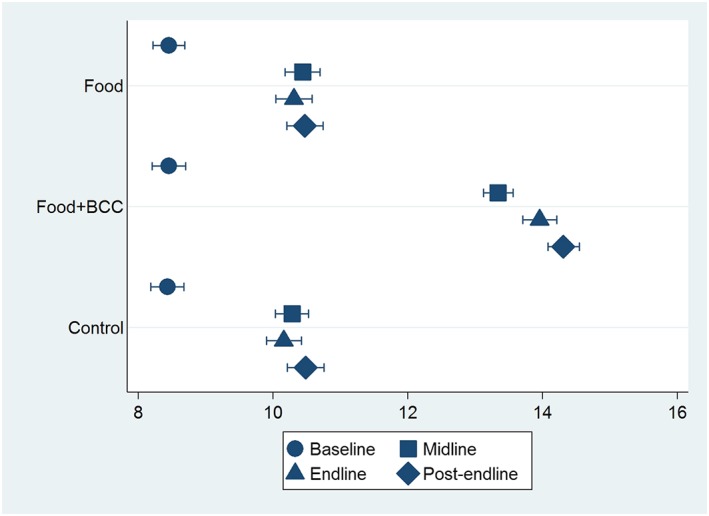
Infant and young child nutrition knowledge scores over survey rounds and by treatment groups—south. Knowledge scores include 18 questions on breastfeeding, sanitation, and other health and nutrition topics. Bars represent 95% confidence intervals

Figure 1 shows the average (and 95% confidence intervals) of the total knowledge score over the four survey rounds for respondents in the north, for each of the three groups in the study (“Cash,” “Cash + BCC,” and “Control”). There is an increase in the total number of correctly answered questions between baseline and midline for all three groups; however, the increase is highest for the “Cash + BCC” group. Between midline and endline and between endline and post‐endline, there is little change in the total number of questions answered correctly for any of the three groups. Figure 2 shows the average (and 95% confidence intervals) of the total knowledge score over the four survey rounds for respondents in the south, again for each of the three groups (“Food,” “Food + BCC,” and “Control”). Again, there is an increase in the number of questions answered correctly between baseline and midline for all three groups, but the increase is largest for the “Food + BCC” group. As with the north, there is minimal change in the total number of questions answered correctly for any of the three groups between midline and endline or between endline and post‐endline. Figures 1 and 2 suggest both that knowledge increases are higher in the BCC intervention groups than in the other groups and that there is persistence of IYCN knowledge.

Table 2 reports the impacts of the TMRI programme on knowledge across rounds through a regression framework, in which statistical significance of these patterns can be assessed. For each of the north (top panel) and the south (bottom panel), three specifications are presented: the total knowledge score at midline, endline, and post‐endline are the outcome variables. Below the regressions for the endline and post‐endline outcome variables, we report *p* values of the differences in coefficients between the regressions. For the north, we report the *p* value of the difference in the “Cash” group coefficient between the midline and the endline in the column for the endline regression and between the endline and the post‐endline in the column for the post‐endline regression. We do the same in the final row of the panel for the difference in the “Cash + BCC” group coefficient between survey rounds. For the south, we report the *p* value of the difference in the “Food” group coefficient between the midline and endline in the column for the endline regression and between the endline and post‐endline in the column for the post‐endline regression. We do the same in the final row of the table for the difference in the “Food + BCC” coefficient between survey rounds.

**Table 2:** Persistence of knowledge across survey rounds

North			
Outcome	Number correct—midline	Number correct—endline	Number correct—post‐endline
	Coefficient/*SE*	Coefficient/*SE*	Coefficient/*SE*
Cash	0.088 (0.244)	0.360 (0.212)	0.491 (0.268)
Cash + BCC	3.251** (0.284)	4.146** (0.199)	4.140** (0.256)
Total number correct—baseline	0.195** (0.032)	0.203** (0.032)	0.219** (0.034)
Constant	8.815** (0.337)	8.190** (0.314)	7.888** (0.348)
Number of observations	1,213	1,213	1,213
*R* ^2^	.313	.423	.370
*p* value for difference in coefficient—Cash		.362	.648
*p* value for difference in coefficient—Cash + BCC		.003	.982

South			
Food	0.154 (0.222)	0.145 (0.193)	−0.016 (0.331)
Food + BCC	3.058** (0.196)	3.794** (0.202)	3.824** (0.296)
Total number correct—baseline	0.154** (0.029)	0.115** (0.035)	0.199** (0.040)
Constant	8.984** (0.275)	9.191** (0.315)	8.807** (0.420)
Number of observations	1,128	1,128	1,128
*R* ^2^	.273	.327	.355
*p* value for difference in coefficient—Food		.966	.628
*p* value for difference in coefficient—Food + BCC		.001	.923

*Note*. Each column and panel represents a separate regression. Outcome is the number of questions answered correctly out of 18 infant and young child nutrition knowledge questions. Sample is restricted to households in which the same respondent answered the infant and young child nutrition questions in all four survey rounds. Estimates are from an ordinary least squares regression. Standard errors are clustered at the village level. The *p* value for difference in coefficients is a chi‐squared test for whether the same coefficient (e.g., Cash or Cash + BCC) differs between midline and endline and between endline and post‐endline. Coefficients were significantly different from 0. BCC = behaviour change communication.

** *p* < .01.

* *p* < .05.

In both regions, the BCC intervention improved knowledge significantly. The total knowledge score is higher among respondents in the “Cash + BCC” group compared to the “Cash” and “Control” groups in the north, and the total knowledge score is higher among respondents in the “Food + BCC” group compared to the “Food” and the “Control” groups in the south. These findings confirm those in Figures 1 and 2. In the north, respondents in the “Cash + BCC” group answer 3.2 more questions correctly at midline, 4.1 more questions correctly at endline, and 4.1 more questions correctly at endline compared to the “Control” group. These effects are statistically significant (*p* < .05). The impact of being in the “Cash” group but not receiving the BCC intervention is not statistically significant (*p* > .05). In the south, respondents in the “Food + BCC” group answer 3.0 more questions correctly at midline, 3.7 more questions correctly at endline, and 3.8 more questions correctly at post‐endline compared to the “Control” group. These effects are statistically significant (*p* < .05). The impact of being in the “Food” group but not receiving the BCC intervention is not statistically significant (*p* > .05).

The difference in programme impacts is statistically significant for the “Cash + BCC” group between midline and endline (*p* < .05) but not between endline and post‐endline (*p* > .05). The difference in programme impacts is statistically significant for the “Food + BCC” group between midline and endline (*p* < .001) but not between endline and post‐endline (*p* ≤ .05).

To assess robustness, we estimated the same regressions as in Table 2 but with the total number of questions correct on the breastfeeding, micronutrients, and WASH questions separately as outcomes. In both the north and in the south, Table 3 shows that knowledge persists across these three sub‐indices. We also re‐estimated the regressions in Table 2 including all of the baseline variables in Table 1 as control variables. Although the point estimates differ slightly, the results are robust to the inclusion of these control variables, and the degree of statistical significance of the point estimates does not differ once the control variables are included. Finally, we also conducted heterogeneity analysis. We look at differences in the following characteristics: the age of the respondent, whether the respondent has had no formal education, the age of the youngest child in the household, and the number of days between the approximate end of the nutrition BCC (May 30, 2014) and the date of administration of the post‐endline survey. There are no significant differences in impact by these characteristics of the household.

**Table 3:** Persistence of knowledge across survey rounds, sub‐indices

North									
	Breastfeeding			Micronutrients			WASH		
Outcome	Midline	Endline	Post‐endline	Midline	Endline	Post‐endline	Midline	Endline	Post‐endline
	Coefficient/*SE*	Coefficient/*SE*	Coefficient/*SE*	Coefficient/*SE*	Coefficient/*SE*	Coefficient/*SE*	Coefficient/*SE*	Coefficient/*SE*	Coefficient/*SE*
Cash	−0.059 (0.079)	−0.020 (0.061)	0.082 (0.060)	−0.033 (0.169)	0.307* (0.147)	0.267 (0.173)	0.180 (0.136)	0.044 (0.126)	0.129 (0.136)
Cash + BCC	0.440** (0.078)	0.557** (0.056)	0.684** (0.060)	1.406** (0.174)	2.136** (0.143)	2.133** (0.176)	1.409** (0.145)	1.451** (0.103)	1.318** (0.118)
Total number correct—baseline	0.023 (0.037)	0.067* (0.033)	0.027 (0.030)	0.173** (0.029)	0.217** (0.030)	0.256** (0.029)	0.071* (0.031)	−0.083** (0.024)	0.106** (0.029)
Constant	1.835** (0.072)	1.709** (0.064)	1.571** (0.057)	5.169** (0.218)	4.639** (0.192)	3.897** (0.209)	2.399** (0.116)	2.453** (0.092)	2.697** (0.096)
Number of observations	1,213	1,213	1,213	1,213	1,213	1,213	1,213	1,213	1,213
*R* ^2^	.085	.118	.145	.165	.303	.272	.201	.258	.189

South									
Food	0.014 (0.061)	0.102 (0.063)	−0.014 (0.063)	0.072 (0.147)	0.004 (0.130)	−0.059 (0.184)	0.070 (0.116)	0.038 (0.112)	0.063 (0.174)
Food + BCC	0.451** (0.059)	0.639** (0.058)	0.742** (0.055)	1.105** (0.136)	1.593** (0.123)	1.801** (0.156)	1.497** (0.115)	1.566** (0.121)	1.258** (0.161)
Total number correct—baseline	0.024 (0.034)	0.082* (0.034)	0.050 (0.032)	0.087** (0.026)	0.073** (0.026)	0.191** (0.031)	0.072* (0.032)	0.114** (0.037)	0.009 (0.031)
Constant	1.687** (0.068)	1.623** (0.065)	1.740** (0.059)	6.064** (0.163)	6.004** (0.163)	4.774** (0.217)	1.919** (0.095)	1.846** (0.093)	2.838** (0.143)
Number of observations	1,128	1,128	1,128	1,128	1,128	1,128	1,128	1,128	1,128
*R* ^2^	.077	.126	.197	.100	.184	.243	.233	.242	.165

*Note*. Each column and panel represents a separate regression. Outcome is the number of questions answered correctly out of 3 breastfeeding knowledge questions (columns 2–4), 11 micronutrients questions (columns 5–7), and 5 WASH questions (columns 8–10). Sample is restricted to households in which the same respondent answered the infant and young child nutrition questions in all four survey rounds. Estimates are from an ordinary least squares regression. Standard errors are clustered at the village level. Coefficients were significantly different from 0. BCC = behaviour change communication.

** *p* < .01.

* *p* < .05.

## 4 DISCUSSION

We find that participants randomly assigned to a nutrition BCC intervention had significantly higher IYCN knowledge 1 year after the intervention began, compared to study participants in treatment groups without BCC. We observe this in both RCTs that we assess. Although participants receiving nutrition BCC also received transfers of cash (in the north) and food (in the south), their IYCN knowledge was higher than that observed in “Cash” only (in the north) and “Food” only (in the south) treatment arms. This suggests that it was the BCC intervention, and not the transfer, that led to increases in IYCN knowledge.

The increase in knowledge from BCC after 1 year was statistically significant. The number of correct answers provided by mothers in the BCC treatment arms increased by 3.2 answers in the north and 3.0 answers in the south, relative to the non‐BCC arms. This is after attending about 48 or 49 BCC sessions on average. Relative to the baseline scores for the control groups, this is equivalent to a 36% increase in the number of correct answers. In the BCC treatment arms, there is a further increase in the number of correct answers during the second year of the intervention (after a further 48 or 49 BCC sessions on average), but the increase is smaller than the change from baseline to midline, rising by 0.9 correct answers in the north and 0.7 correct answers in the south. Results from the post‐endline survey showed that these gains in knowledge persisted. No significant decline in IYCN knowledge was observed 6 to 10 months after the BCC intervention ended. The effects do not differ when we separate IYCN knowledge topics into those of breastfeeding, micronutrients, and WASH. There are no significant differences in effects based on the respondent's age or education level, the age of the youngest child in the household, or the amount of time that had elapsed between the final BCC session and post‐endline surveys.

This improvement and then persistence in knowledge may be due to a number of factors. It is possible that the frequency of the BCC sessions enabled high uptake and retention of information. Involvement of other household members may have encouraged further discussion of these topics at home and made it easier not only to learn but also to retain the information. It is also possible that the home follow‐up visits by CNWs when sessions were missed provided an opportunity for participants to ask questions about and seek clarifications of material that was covered in the group BCC sessions. Our data do not allow us to identify the separate effects of these mechanisms. Further, it may be the combination of these factors that were important for this sustained knowledge gain.

There are three important caveats for interpreting our results. First, our measure of knowledge focuses heavily on the nutrient content of foods, paying less attention to other aspects of knowledge such as an understanding of the consequences of nutrient deficiency. Second, knowledge gain and persistence do have limitations. Depending on food availability, cultural norms, and potentially other factors, knowledge gain and retention do not necessarily translate into behaviour change. Knowledge is necessary, but is not sufficient. Lastly, there is no treatment arm that is BCC only; we are attributing the effects on knowledge to the BCC because there were no impacts in the non‐BCC intervention arms. Our results should thus be interpreted with this caveat in mind.

Our study has several strengths. Our analysis is based on cluster RCTs, which alleviates concerns that confounding factors could be driving the effects. Our results are therefore causal rather than associational. We obtain similar results from two RCTs fielded in two different parts of rural Bangladesh. We have data on IYCN knowledge from the same mothers at multiple time points, prior to intervention as well as from 13 to 34 months after the intervention commenced. Our results remain robust to including baseline household characteristics and respondent characteristics in the analysis.

Our study also has weaknesses. In the south RCT, there were minor imbalances in baseline characteristics between the treatment groups. However, controlling for these baseline characteristics in the analysis do not change the results, and so, we do not believe this should be a cause for concern. There was one factor in the north and in the south that affected attrition. However, the magnitudes of their effect on attrition were extremely small, and we do not believe that they would bias our estimates. Finally, as noted above, the data do not allow us to comment on the precise mechanisms through which these effects occurred; we cannot be certain whether the effects were due to the frequency of BCC sessions, the involvement of husbands and/or mothers in law, in‐home follow‐up visits, or some combination of these.

Although we are able to examine whether gains in IYCN knowledge persist 6–10 months after the BCC interventions ended, it would be of interest to see whether the persistence continues over a longer period of time. It would also be useful to know whether these knowledge gains could be achieved through a shorter duration of BCC activities or through activities that were less intense and less costly. Such an assessment should also consider whether a shorter duration, or a less intensive approach, affected the extent to which caregivers act on this knowledge and whether the shorter duration/reduced intensity affected impact on measures of nutritional status. Future work could examine these issues.

Our core finding that knowledge gained through nutrition BCC is retained after the BCC ends has implications for the design and implementation of such interventions in Bangladesh and elsewhere. They suggest that the costs of well‐implemented, intensive BCC can be justified by the fact that the gains in IYCN knowledge persist after the training ends. This implies that children born after this intervention ended may well benefit from their mothers' improved IYCN knowledge. This also means that studies measuring the impact of such programmes may underestimate impacts if the degree of persistence of impacts remains unaccounted for. Subject to the caveats described above, our results are suggestive that many of these gains can be achieved through 12 months of this type of intensive, well‐delivered BCC that includes follow‐up home visits for missed sessions and facilitates involvement of other household members.

## CONFLICTS OF INTEREST

The authors declare that they have no conflicts of interest.

## Supporting information


**Table S1**: Infant and Young Child Nutrition Knowledge Questions, Responses, and Correct Answers
