# Supplementary material for: Nutrition behaviour change communication causes sustained effects on IYCN knowledge in two cluster‐randomised trials in Bangladesh
Source: Matern Child Nutr. 2017 Aug 7;14(1):e12498. doi: 10.1111/mcn.12498 (PMC5763316; doi:10.1111/mcn.12498)
Supplement: Supplementary file 1 — Table S1: Infant and Young Child Nutrition Knowledge Questions, Responses, and Correct Answers [file MCN-14-e12498-s001.docx]

Supplementary Material

**Table S1: Infant and Young Child Nutrition Knowledge Questions, Responses, and Correct Answers**

|  | **Question** | | **Answer code** | **Correct Answer(s)** |
| --- | --- | --- | --- | --- |
| 1 | How often should a baby breastfeed? | | Whenever baby wants 1  When you see the baby is hungry 2  When the baby cries 3  Other (specify) 4  Don’t know 88 | 1 or 2 |
| 2 | If a mother thinks her baby is not getting enough breast milk, what should she do? | | Breastfeed more often/more frequently..1  Give other liquids/foods… …...2  Mother needs to drink more water …...3  Mother needs to eat more food …...4  Other (specify) …...5  Don’t know ….88 | 1, 3, or 4 |
| 3 | Do you think that infants under 6 months of age should be given water if the weather is very hot? | | Yes 1  No 2  Don’t know 88 | 2 |
| 4 | Name one thing that can happen to children if they do not get enough iron (either in their diet or via iron supplements) | | Impaired learning 1  Impaired development 2  Lower height 3  Weakened immune defense 4  Feel tired 5  Become anemic 6  Other (specify) 7  Don’t know 88 | 1, 2, 4, 5, or 6 |
| 5 | What seasoning (food item) is often fortified with iodine (a nutrient important for brain development)? | | Salt 1  Other (specify) 2  Don’t know 88 | 1 |
| 6 | How should you wash your hands: | | | |
|  | 6.1 | Before feeding myself and child | By soap 1  By ash 2  By mud 3  Only water 4 | 1 |
|  | 6.2 | After using toilet |  | 1 |
|  | 6.3 | What you use for washing your hand after cleaning a child who has defecated |  | 1 |
|  | 6.4 | What you use for washing your hand other any time |  | 1 |
| 7 | Generally how should you clean vegetables? | | Cut into small pieces and then wash in water…………………………………….1  Wash the whole vegatable and then cut into pieces……………………………………2  Combination.............................................3  Others ........4 | 2 |
| 8 | Which of these three foods is a rich source of Vitamin A? | | Green Leafy Vegetables………………1  Rice……………………………………2  Pulses………………………………….3  Don't know……………………………4 | 1 |
| 9 | Which of these three foods is a rich source of Vitamin A? | | Wheat………….………………………1  Cooking oil……………………………2  Mango (ripe)…………………………..3  Don't know…………………………….4 | 3 |
| 10 | Which of these three foods is a rich source of Vitamin A? | | Maize………….………………………1  Mola fish………………………………2  Coriander……………………………...3  Don't know……………………………4 | 2 |
| 11 | Which of these three foods is a rich source of iron? | | Liver………….………………………1  Rice………………………………..…2  Banana……….……………………….3  Don't know……………………………4 | 1 |
| 12 | Which of these three foods is a rich source of iron? | | Amaranth………….…………………1  Potato………...………………………2  Puti fish…….………………………...3  Don't know………………...…………4 | 1 |
| 13 | Which of these three foods is a rich source of iron? | | Colocasia leaves………….……………1  Butter…………………..………………2  Snake head fish…….……….……….…3  Don't know………………………..……4 | 1 |
| 14 | Which of these three foods is a rich source of Vitamin C? | | Meat…………….……………………1  Guava……………...…………………2  Fish…….……….…..………………...3  Don't know……………………………4 | 2 |
| 15 | Which food prevents you from having goiter? | | Iodized salt………………………………1  Leafy vegetables…………………………2  Guava…….……….……………………...3  Don't know………………………………4 | 1 |
